# The association of meteorological factors and mortality in rural Bangladesh, 1983–2009

**DOI:** 10.3402/gha.v5i0.19063

**Published:** 2012-11-23

**Authors:** Wietze Lindeboom, Nurul Alam, Dilruba Begum, Peter Kim Streatfield

**Affiliations:** 1 Centre for Population, Urbanization and Climate Change, Icddr,b, Dhaka, Bangladesh; 2 Cardialysis, Rotterdam, The Netherlands

**Keywords:** climate change, mortality, Matlab

## Abstract

**Introduction:**

While the association of weather and mortality has been well documented for moderate climate zones, little is known about sub-tropical zones, particularly Bangladesh. This study aims to assess the short-term relationship of temperature and rainfall on daily mortality after controlling for seasonality and time-trends. The study used data from Matlab, Bangladesh, where a rigorous health and demographic surveillance system (HDSS) has been operational since 1966.

**Material and methods:**

Matlab HDSS data on mortality and population for the period 1983–2009 were used. Weather data for the same period were obtained from a nearby government weather station. Time series Poisson regression with cubic spline functions was applied allowing for lagged effects of weather and extreme weather events on mortality, and controlling for time trends and seasonal patterns. Analysis was carried out using R statistical software.

**Results:**

Both temperature and rainfall showed strong seasonal patterns, explaining a significant part of mortality in all age groups. After adjusting for seasonality and trend, mortality and temperature show a U-shaped pattern; below a temperature of around 29°C, a decrease in temperature resulted in an increase in mortality, whereas above 29°C, increased temperature resulted in increased mortality. The strongest negative mortality temperature association was observed in the elderly (5.4% increase with every 1°C decrease in temperature at temperatures below 23°C), and the opposite trend was observed in the age groups 1–4 and 5–19 years old. At aggregate level, the rainfall–mortality association is statistically weak. However in the age group 5–19, a 0.6% increase in mortality per 1 mm additional rainfall was found, at rainfall levels over 100 mm per day. Multivariate analysis showed high mortality risks for women aged 20–59 years of age during cyclone episodes.

**Discussion:**

Weather and extreme weather were associated with mortality with differential impacts in age and sex sub-groups. Further studies should investigate these findings more closely and develop policy recommendations targeted at improving public health and protecting population groups susceptible to environmental stressors.

Extreme climate events, such as extreme daily temperatures, extreme daily rainfall amounts, large areas experiencing unusually warm temperatures, or storms, are predicted to increase in frequency and duration with ongoing climate change (1). As per the Bangladesh Bureau of Statistics, the number of major cyclones was 13 during 1897–1947 and rose to 51 during the next 50 years. Bangladesh Meteorological Department (BMD) data on minimum and maximum temperatures observed in 1950–2010 showed an increasing trend and the increase was faster for minimum temperature (2). Frequency of extreme events such as cyclones with wind speed of >200 km/hour and heavy rainfall in pre-monsoon has increased in recent years. The rate of wet days is high in the North-east and has increased in the South-east regions of Bangladesh. Both temperature and rainfall data show signs of climate change. Increase in extreme events and vulnerability to climate change raises the importance of more accurate forecast and warning at local level for mitigation and adaptation of climate effects.

In recent decades, several devastating heat weaves have caused large health consequences in urban areas across the globe (3). The effects of heat waves on morbidity were less obvious than on mortality in developed countries. Heat waves were associated with increased mortality amongst the elderly in developed countries, while cold waves were associated with increased mortality of children and the elderly in developed and developing countries (4, 5).

Studies examining health effects of extreme climate events (hot or cold) were far less in developing countries – the main reason being the lack of reasonable quality health data over a longer period of time. One study examined seasonal patterns of deaths in Matlab, Bangladesh while another study reported that daily mortality increased with low temperatures in the preceding weeks and no association between high temperature and daily mortality during 1994–2002 (5, 6). These studies were limited to examine associations between mortality and temperature, excluding rainfall and extreme weather events.

## Objectives

The objectives are to investigate the short-term association between weather and day-to-day causes of mortality in Matlab, a rural area of Bangladesh, where a rigorous demographic surveillance has been operating since 1966. Disaggregating the effect by age and gender is another objective of the study.

## Methods and data

This study used high quality longitudinal vital registration data in Matlab and daily weather data from a nearby weather station of the metrological department of the government of Bangladesh. The health and demographic surveillance system (HDSS) maintained by the International Centre for Diarrhoeal Disease Research, Bangladesh (icddr,b) in Matlab, recorded vital events, that is, births, deaths, and migrations since 1966 and marriage and marital disruptions from 1975, visiting households monthly until 2000 and bimonthly thereafter.

Meteorological data (daily minimum and maximum temperature, rainfall, and relative humidity) for 1983–2009 from Chandpur district, located 10 km from Matlab town, were provided by the BMD. Missing values were replaced through linear interpolation.

Counts of deaths and population at risk each day were linked with daily weather data to examine the seasonal patterns of temperature, rainfall and mortality and also to estimate effects of temperature and rainfall on mortality of different age groups and sexes, accounting for long terms trends and seasonal patterns. Information on cyclones in Bangladesh was obtained from Banglapedia and SMRC No. 1. (7, 8).

## Statistical analysis

The association between daily temperature (minimum, maximum, and mean), rainfall and mortality was examined using graphs followed by generalized additive Poisson regression models with cubic spline functions, allowing for over dispersion, using the following general formula:

$$\begin{array}{l} {\text{Mortality}}_{\text{t}}\sim {\text{Poisson(mean}}_{\text{t}}\text{)}\\ {\text{log(mean}}_{\text{t}}{\text{) = b}}_{\text{0}}{\text{+ s(time}}_{\text{t}}{\text{,df = 6 per year) + s(temperature}}_{\text{t}}{\text{,df = 10) + s(rainfall}}_{\text{t}}\text{,df = 10)} \end{array}$$

Where t denotes time, s denotes a natural cubic spline function and df denotes the degrees of freedom of the natural cubic spline function.

For linear segmented approximation of the weather-mortality relationships separate slopes of the weather variables below the 25th percentile, between the 25th and 75th percentile, and above the 75th percentile were determined. When appropriate alternative cut-off points were used, dummy variables were used to indicate occurrences of extreme events, that is, cyclones. The indicator variables, public holidays, and festivals were incorporated into the model to estimate variation in mortality relating to change in behavior.

Models were fitted to daily temperature and rainfall of the day of death and of mean temperature and rainfall up by 21 days prior to the day of death (lags 0–21) to identify the effects of longer periods with high or low temperatures or high or low rainfall. Combined time trend and seasonal pattern were included in cubic splines with six un-penalized degrees of freedom for seasonal pattern and trend, per year. The exposure-response to meteorological factor was penalized allowing a maximum of 10 degrees of freedom.

## Results

During the 9,862 days of observation, equivalent to 27 years, 48,238 deaths were registered, which was on average 4.9 deaths per day, with a peak of 59 deaths registered on February 19, 2005, due to a launch (large commuter boat carrying passengers) accident. Infants (23.3%) and elderly (42.3%), defined as age 60 and above, account for over 65% of all deaths. During the observation period, the population increased from 190,183 on the first day of 1983 to 225,002 on the last day, that is, December 31, 2009. Between January 1, 1983, and December 31, 2009, the lowest minimum temperature observed was 8.6°C, and the highest maximum was 37.8°C, with mean temperatures ranging from 13.1°C to 32.6°C. Average daily rainfall was 5.8 mm with a peak rainfall of 334 mm in a single day. In Matlab, and Bangladesh in general, three seasons can be distinguished. 1) A calendar starts and ends with a winter season (November–February), with a mean temperature±standard deviation of 21.6°C±2.8, and 0.6 mm±5.4 daily rainfall. 2) The hot and dry season runs from March to May with a second period in October, with a mean temperature of 27.9°C±2.1 and an average daily rainfall 5.2 mm±15.1. 3) The middle of a calendar year is characterized by hot and humid weather, temperatures range from 22.9°C to 32.6°C, with a mean of 28.9°C±1.3 and an average daily rainfall of 11.6 mm±22.0. Between 1983 and 2009, Bangladesh was hit 14 times by major cyclones where the centre of the last two major cyclones, reported in 2007 and 2009, did not pass through the Matlab research area.

**Fig. 1:**
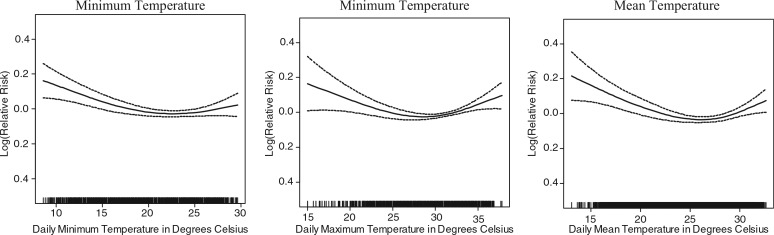
Association of mortality with minimum, maximum and mean temperature, after adjusting for trend and seasonality.

To assess the association between weather, weather extremes and mortality, daily minimum and maximum temperatures and rainfall measures were used. Analysis showed that the mean (average of the daily minimum and maximum) temperatures exhibited stronger associations with mortality across the temperature range than minimum and maximum temperature. Fig. 1 shows the smoothed functions of the relative overall mortality risk in association with daily minimum, maximum and mean temperature, after adjusting for trend and seasonality. In Table 1 the adjusted linear approximations of the above mentioned smoothed functions are given for temperatures below the first quartile (lowest 25%), between the first and last quartiles and for temperatures above the last quartile (above the 75th percentile). The smoothed functions showed only minor differences between the three temperature measures in model fit statistics. However, mean daily temperature showed slightly stronger association in the linear approximation over the first, second to third and fourth quintile, with a linear negative relationship resulting in a 1.4% increase in mortality with every 1°C decrease in mean temperature, at temperatures below 29.2°C, and a positive relationship between mortality and mean temperature, at temperatures over 29.2°C, with a 0.2% increase in mortality with every 1°C increase in mean temperature. For further modeling, and determination of optimal time-lag between temperature and mortality, mean daily temperature was be used.

**Table 1:** Linear approximation of the association of mortality with minimum, maximum and mean daily temperature, after adjusting for trend and seasonality

	<25% (first quartile)			25%–75% (2nd and 3rd quartile)		>75% (last quartile)				
					
Daily temperature	Temperatures below	Change (%)	95% Confidence Interval	Change (%)	95% Confidence Interval	Temperatures above	Change (%)	95% Confidence Interval	Deviance explained	GCV^2^
Maximum	28.2	−**0.7** **1**	(−1.5, −0.0)	−**0.8**	(−1.5, −0.2)	32.9	**0.2**	(0.1, 0.3)	26.9%	1.1285
Minimum	18.0	−**1.1**	(−1.8, −0.3)	−**1.1**	(−1.7, −0.6)	25.9	**0.1**	(0.0, 0.2)	26.8%	1.1291
Mean	23.3	−**1.4**	(−2.2, −0.6)	−**1.4**	(−2.0, −0.7)	29.2	**0.2**	(0.1, 0.3)	26.8%	1.1283

1 Change less than 0% indicates decrease in mortality risk with increase in temperature, or at lower temperature ranges, decrease in temperature results in increased mortality risk.

2 GCV Geometric Coefficient of Variation gives an indication of the ‘variance – mean ratio; higher values indicate higher level of variation.

**Fig. 2:**
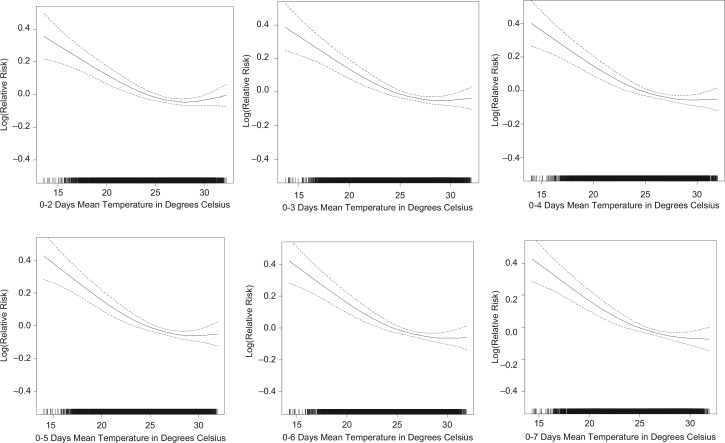
Association of mortality with mean temperature at different time lags, after adjusting for trend and seasonality.

**Fig. 3:**
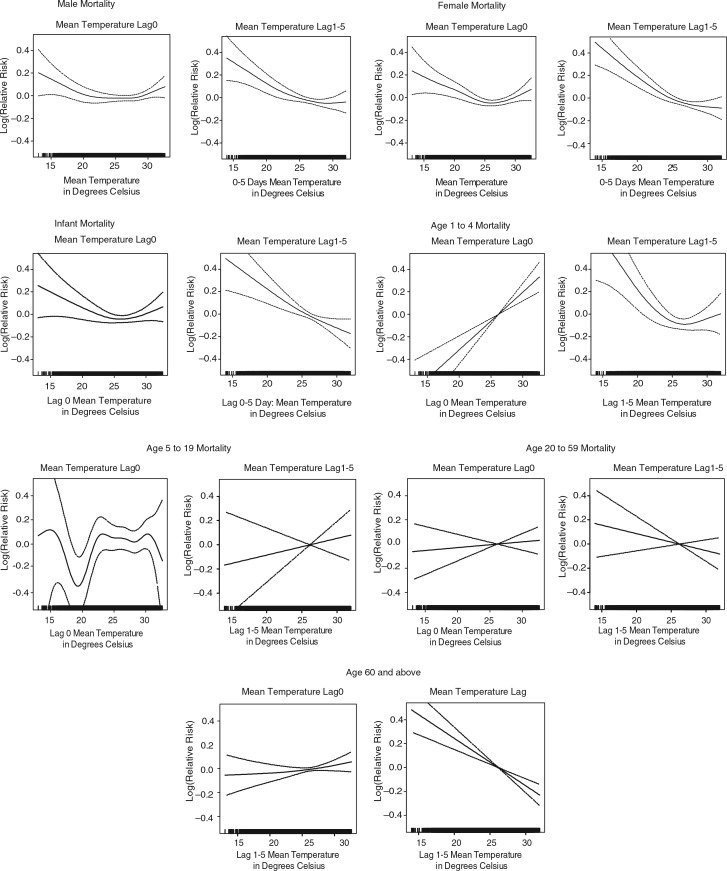
Association of mortality with lag 0 and lag 1–5 mean temperature for different strata, after adjusting for trend and seasonality.

Associations between mean temperature at different time lags and mortality, after adjusting for trend and seasonality, are presented in Fig. 2. Assessment of the graphs and the relative risks presented in Table 2 shows that the lag 1–5 temperature model better predicts mortality at temperature below the 75th percentile (2.4 and 2.3% increase in mortality per 1°C decrease in temperature), while shorter time lags show stronger associations of mortality and mean temperature at temperatures above the 75th percentile. Model fit statistics only slightly differ between the different time lags – deviance ranged from 26.8% to 27.0% and the geometric coefficient of variance (GCV) ranged from 1.1259 to 1.1292. To capture the short term temperature effect at higher temperatures and the longer term effect of increased mortality at lower temperatures simultaneously, lag 0 and lag 1–5 mean temperatures were used to assess the association between temperature and mortality in different age groups and for men and women. In general, the stratified models showed a positive association between mortality and daily temperature, whereas the lag 1–5 models demonstrate a negative association between temperature increase and mortality (Fig. 3). Age group 5–19, with a relatively small number of deaths, showed an irregular association of temperature and mortality. The combined segmented linear approximations association of lag 0 and lag 1–5 mean temperatures are shown in Table 3. Elderly, aged 60 years and above, seem to be most effected at lower temperatures, with a 5.4% increase in mortality with every 1°C decrease in temperature, at temperatures below 23°C. Though not statistically significant, the age groups 1–4 years and 5–19 years showed the opposite trend.

**Table 2:** Linear approximation of the association of mortality with mean temperature at different time lags, after adjusting for trend and seasonality

	<25% (first quartile)			25%–75% (2nd and 3rd quartile)		>75% (last quartile)				
					
Lag	Temperatures below	Change (%)	95% Confidence Interval	Change (%)	95% Confidence Interval	Temperatures above	Change (%)	95% Confidence Interval	Deviance explained (%)	GCV
0–1	23.4	−**1.8**	(−2.6, −1.0)	−**1.8**	(−2.5, −1.2)	29	**0.20**	(0.1, 0.3)	26.9	1.1268
0–2	23.4	−**2.0**	(−2.8, −1.2)	−**2.0**	(−2.7, −1.3)	29	**0.20**	(0.1, 0.3)	27	1.1259
0–3	23.4	−**1.9**	(−2.8, −1.1)	−**2.0**	(−2.7, −1.3)	29	0.10	(0.0, 0.2)	27	1.1259
0–4	23.4	−**2.2**	(−3.0, −1.3)	−**2.2**	(−2.9, −1.4)	29	0.10	(0.0, 0.2)	27	1.1262
0–5	23.4	−**2.4**	(−3.3, −1.5)	−**2.3**	(−3.1, −1.6)	29	0.10	(0.0, 0.2)	27	1.1261
0–6	23.4	−**2.3**	(−3.2, −1.4)	−**2.3**	(−3.0, −1.5)	29	0.10	(0.0, 0.2)	27	1.1263
0–7	23.4	−**2.1**	(−3.0, −1.1)	−**2.1**	(−2.9, −1.3)	29	0.10	(0.0, 0.2)	27	1.1264
0–8	23.3	−**2.2**	(−3.2, −1.3)	−**2.2**	(−3.0, −1.4)	28.9	0.00	(−0.1, 0.1)	27	1.1268
0–9	23.3	−**2.2**	(−3.2, −1.3)	−**2.2**	(−3.0, −1.4)	28.9	0.00	(−0.1, 0.1)	27	1.1269
0–10	23.3	−**1.9**	(−2.9, −0.9)	−**1.9**	(−2.7, −1.1)	28.9	0.00	(−0.1, 0.1)	27	1.1269
0–14	23.4	−**2.1**	(−3.1, −1.1)	−**2.0**	(−2.9, −1.2)	28.9	0.00	(−0.1, 0.1)	27	1.1279
0–21	23.4	−**1.3**	(−2.3, −0.2)	−**1.3**	(−2.2, −0.4)	28.9	−0.10	(−0.2, 0.0)	26.8	1.1292

Note: Statistically significant (0.05 level) relative risk estimates are marked in bold.

**Table 3:** Linear approximation of the association of mortality with combined lag 0 and lag 1–5 mean temperature for different strata, after adjusting for trend and seasonality

	25 percentile		25–75 percentile		75 percentile	
			
	Change (%)	95% CI	Change (%)	95% CI	Change (%)	95% CI
Sub groups						
Male	−**3.2**	(−5.1, −1.3)	−**2.9**	(−4.6, −1.2)	**0.2**	(0.0, 0.4)
Female	−**2.2**	(−4.3, −0.2)	−**2.3**	(−4.1, −0.5)	0.2	(−0.1, 0.4)
						
Age groups						
Infants	−2.4	(−5.2, 0.5)	−2.3	(−4.8, 0.3)	0.2	(−0.2, 0.5)
1–4	2.9	(−1.1, 7.0)	2.9	(−0.5, 6.6)	**0.4**	(0.0, 0.9)
5–19	3.6	(−1.8, 9.2)	3.7	(−1.1, 8.7)	−0.2	(−0.8, 0.4)
20–59	−1.6	(−4.8, 1.6)	−1.7	(−4.4, 1.2)	0.0	(−0.4, 0.4)
60+	−**5.4**	(−7.4, −3.5)	−**5.3**	(−7.0, −3.6)	0.2	(−0.1, 0.4)

Note: Statistically significant (0.05 level) relative risk estimates are marked in bold.

**Fig. 4:**
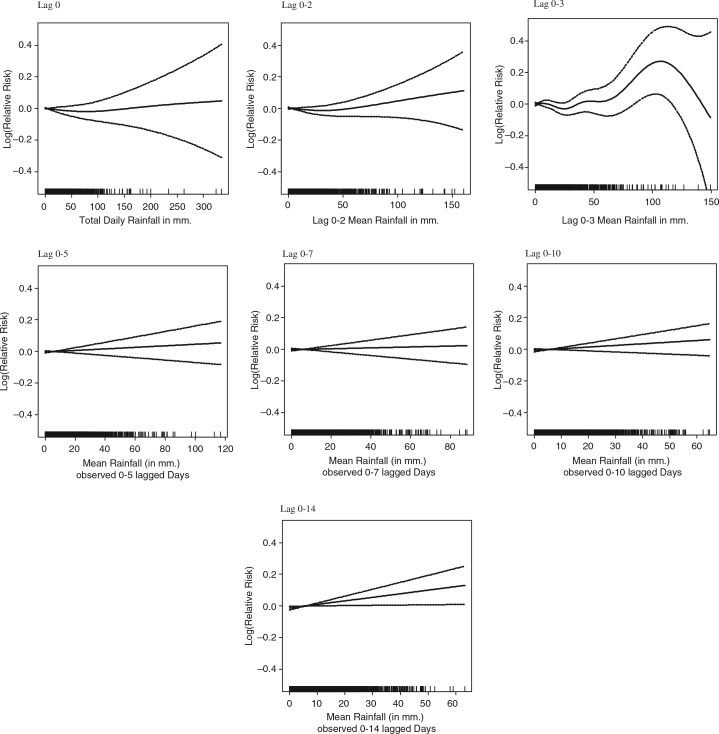
Association of mortality with rainfall at different time lags, after adjusting for trend and seasonality.

**Fig. 5:**
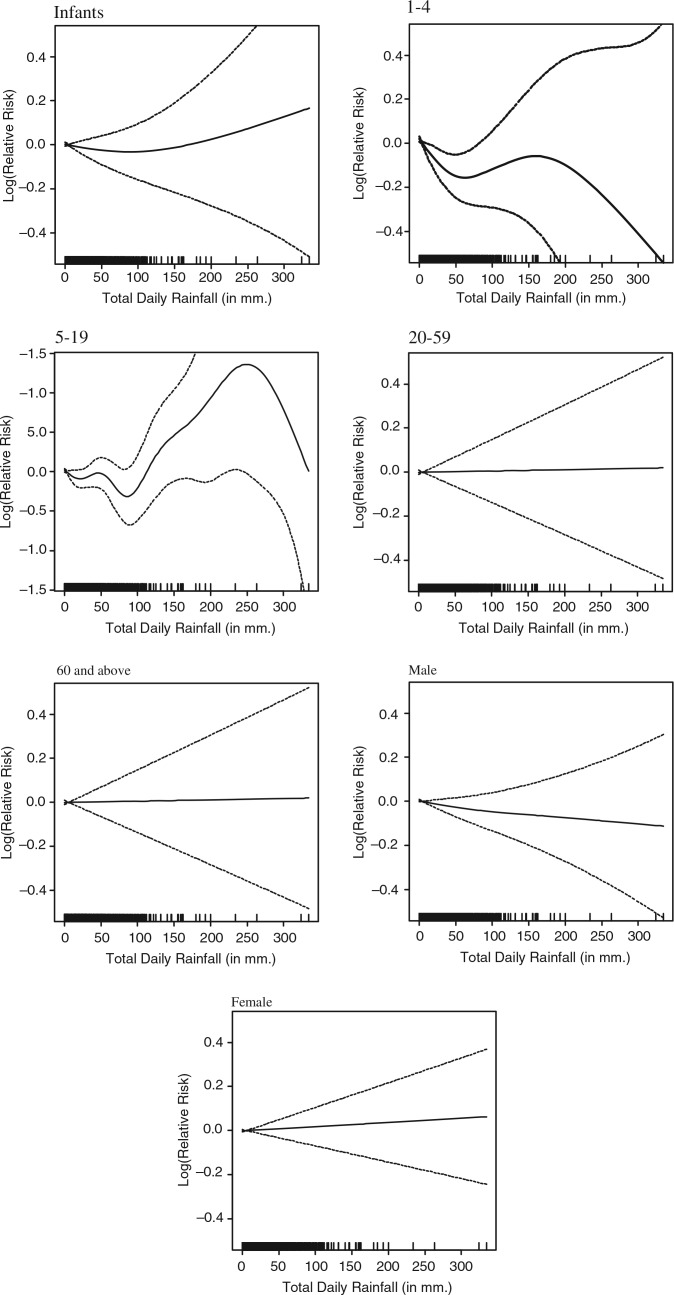
Association of mortality with rainfall for different strata, after adjusting for trend and seasonality.

The average daily rainfall of 5.8 mm is the result of a skewed rainfall pattern, with an average 251 days per year with rainfall below 1 mm. For the linear approximation of rainfall models, the 75 and 95% cut-off points were chosen, corresponding with 3 mm and 34 mm rainfall for lag 0. Associations between rainfall at different lags and mortality were weak – only lag 0–3 rainfall deviated from the other presented models (see Fig. 4). None of the segmented linear associations were statistically significant at aggregate level. Moving the upper cut-off point for lag 0 rainfall up from 34 to 100 mm (99.6%) resulted in statistically significant slopes between 3 mm and 100 mm, with a 0.1% reduction in mortality per 1 mm additional rainfall, and above 100 mm a 0.1% increase in mortality per 1 mm increase in rainfall (Table 4). When studying age groups (Table 5 and Fig. 5), a pronounced and statistically significant association was found in the age group 5–19 years when daily precipitation was above 100 mm (0.6% mortality increase per 1 mm additional rainfall).

**Table 4:** Linear approximation of the association of mortality with rainfall at different time lags, after adjusting for trend and seasonality

	Rainfall below 3 mm		Rainfall between 3 and 100 mm		Rainfall above 100 mm	
			
Lag	Change (%)	95% CI	Change (%)	95% CI	Change (%)	95% CI
0	−1.2	(−4.0, 1.7)	−**0.1**	(−0.2, 0.0)	**0.1**	(0.0, 0.2)
0–2	0.0	(−0.2, 0.1)	0.0	(−0.1, 0.1)	0.1	(−0.1, 0.3)
0–3	0.0	(−0.1, 0.2)	0.0	(−0.1, 0.1)	0.1	(−0.1, 0.3)
0–5	0.1	(−0.1, 0.2)	0.0	(−0.1, 0.1)	0.2	(−0.2, 0.5)
0–7	0.0	(−0.2, 0.2)	0.0	(−0.2, 0.2)	0.2	(−0.2, 0.6)
0–10	0.1	(−0.1, 0.3)	0.0	(−0.1, 0.2)	0.3	(−0.2, 0.8)
0–14	0.0	(0.0, 0.0)	0.0	(0.0, 0.1)	0.0	(0.0, 0.1)

Note: Statistically significant (0.05 level) relative risk estimates are marked in bold.

**Table 5:** Linear approximation of the association of mortality with rainfall for different strata, after adjusting for trend and seasonality

	Rainfall below 3 mm		Rainfall between 3 and 100 mm		Rainfall above 100 mm	
			
	Change (%)	95% CI	Change (%)	95% CI	Change (%)	95% CI
Sub groups						
Male	−1.3	(−5.2, 2.7)	−0.1	(−0.3, 0.0)	**0.2**	(0.0, 0.3)
Female	0.0	(0.0, 0.0)	0.0	(0.0, 0.1)	0.0	(0.0, 0.1)
Age groups						
Infants	−1.3	(−7.1, 4.9)	−0.1	(−0.3, 0.0)	0.2	(−0.1, 0.4)
1–4	1.7	(−5.6, 9.5)	−0.2	(−0.4, 0.0)	0.1	(−0.1, 0.4)
5–19	−5.6	(−15.0, 5.0)	−0.3	(−0.6, 0.0)	**0.6**	(0.2, 0.9)
20–59	−3.3	(−9.4, 3.3)	−0.2	(−0.4, 0.0)	0.2	(−0.1, 0.5)
60+	0.1	(−0.1, 0.2)	0.0	(−0.1, 0.1)	0.2	(−0.2, 0.5)

Note: Statistically significant (0.05 level) relative risk estimates are marked in bold.

To assess joint association of weather variables with mortality, models were built that included lag 0, lag 1–5 mean temperature, and lag 0 mean rainfall. Occurrence of cyclones was included as an exponent of extreme weather events. When statistically significant, national festivals, the Holy month of Ramadan and related feasts were included in the model as co-variates. To increase comparability, identical models were used in the different age groups and for both sexes. In none of the age groups or sex groups, the national festivals, the Holy month of Ramadan and related feasts showed significant associations with mortality. The occurrence of cyclones was associated with a 34% (0.0%, 77%) increase in mortality in the age group 20–59 and for 24% for women (95% CI 4%, 48%), resulting in an increased mortality risk of 58% for women in the age group 20–59, with 95% confidence limits of 10 and 124%.

**Fig. 6:**
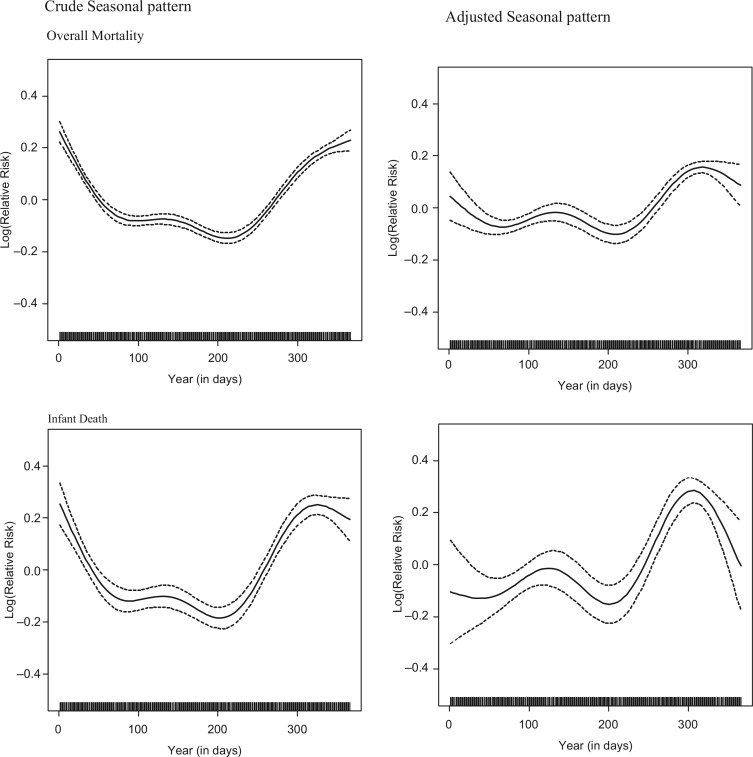

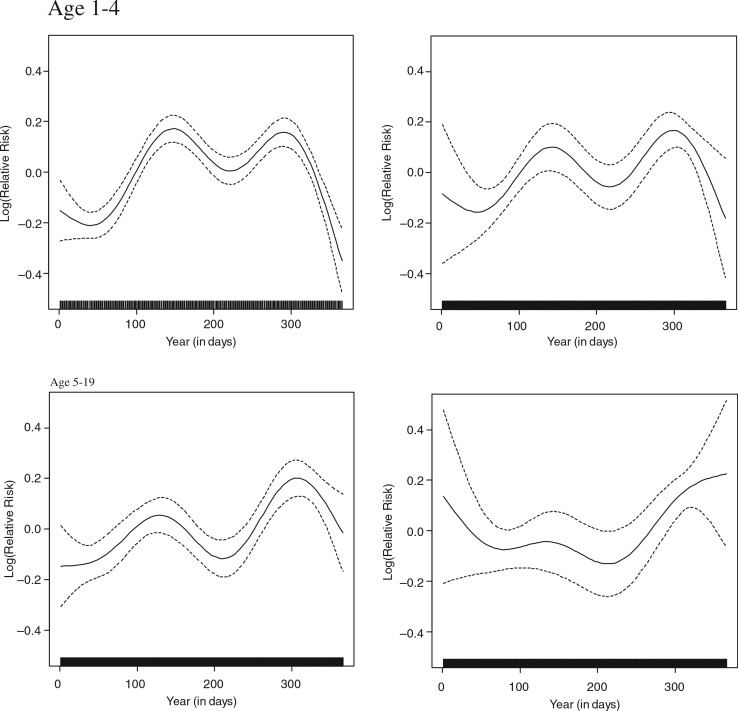

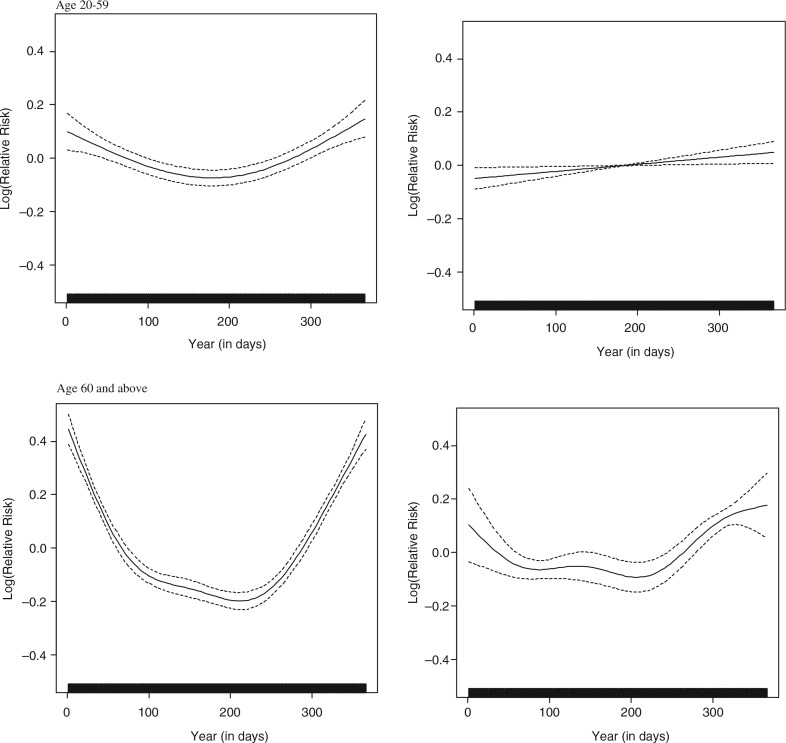

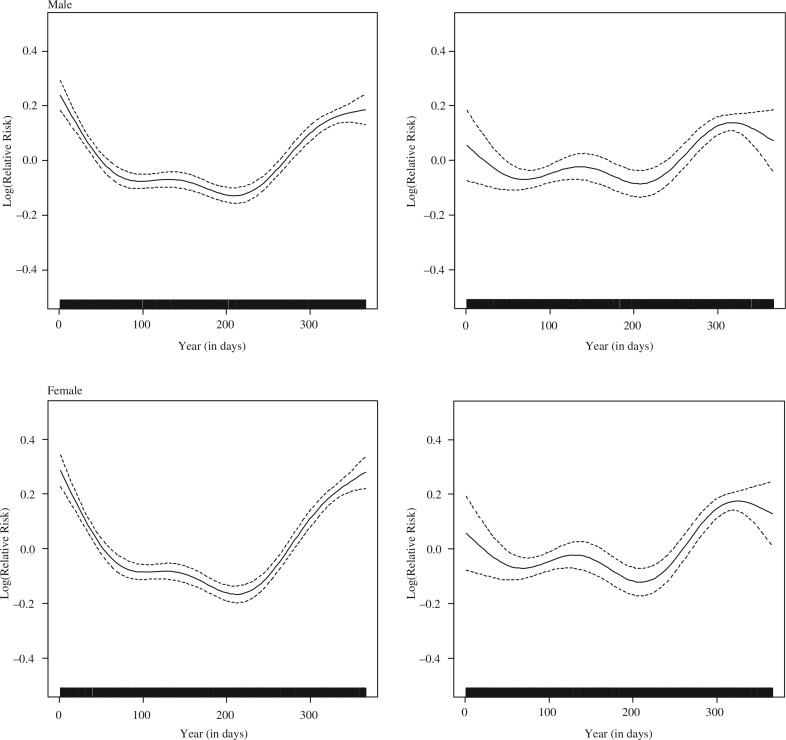
Seasonality of mortality, adjusting for trend only, and adjusting for weather variables and trend.

Mortality in the Matlab surveillance area shows overall weak associations with rainfall (in all but age groups 15–19), and stronger negative association with temperature. Temperature and rainfall both show peaks around the middle of the year, consequently mortality rates will be higher at the beginning and towards the end of the calendar year. Consistent with temperature associations, overall mortality shows U-shaped seasonal pattern with higher mortality risks during the first two and last 2 months of the year, and the lowest risk in June–August. With the exception of the age groups 1–4 and 5–19 years, the other age groups follow the same seasonal pattern. The age groups 1–4 and 5–19 years both show a bi-modal pattern, the first peak accruing around April-May and the second peak around October–November. The left hand panel of Fig. 6 shows the seasonal pattern of mortality risks before adjusting for weather covariates; the right-hand panel shows the seasonal pattern for log transformed relative mortality risks for the different strata after adjusting for weather co-variates. The graphs in Fig. 6 clearly show that part of the seasonal pattern in overall mortality is removed by the weather covariates, which means that short-term temperature and rainfall shape seasonality, but that there are also other unknown factors that are important determinants of seasonality. The adjusted seasonal patterns for different age groups range from little change in the age group 1–4 years to an almost reversed pattern in the age groups of 0 years and of 5–19 years; the infant mortality risk around the pre-monsoon period becomes more pronounced after adjusting for weather effects; the peak around October–November coincides with higher daily birth rates during these months and associated larger numbers of neonatal deaths.

## Discussion

In Bangladesh, both births and deaths have striking seasonal patterns and so does temperature and rainfall (2, 6). This study revealed, after adjusting for trend and seasonal patterns in mortality, a marked increase in overall mortality at lower temperature with age and sex effects in the Matlab area. In particular, deaths of infants and the elderly (aged 60 years and older) were more frequent in periods of lower temperature compared to days with higher average temperature. The result of excess mortality of infants and elderly in the cold period is consistent with the findings of the previous studies that marked higher mortality in perinatal age and elderly (65 years and older) in winter months even as the level of mortality has declined (5, 6).

With the exception of the age groups 1–4 and 5–19, the other age groups followed the same seasonal pattern. The age groups 1–4 and 5–19 years both showed a bi-modal pattern, the first peak accruing around April–May and the second peak around October–November. In many tropical developing nations, the peak of deaths in 1–4 years was in the summer (9). Seasonality became weaker, after adjusting for the weather co-variates; the U-shaped seasonal pattern of infant mortality changes into a bi-modal pattern, with elevated mortality risks around April–May and higher peaks starting in October. For the age group 5–19, the change is in the opposite direction, from a bi-modal crude seasonality pattern to a U-shaped adjusted seasonality of mortality.

Mortality in the age group 20–59 year exhibited a weaker seasonal pattern compared to the other age groups. Though the cyclones Sidr on November 15, 2007, and Aila on May 25, 2009, did not hit the study area hard, women and not men aged 20–59 experienced extremely high mortality risks (58% increase) during the cyclone episodes. We did not find an explanation as to why only this specific group experienced an increased mortality risk during cyclones. However we may asssume that women, especially those with younger children are less mobile and therefore more susceptible.

Mortality in the Matlab surveillance area shows overall weak associations with rainfall, and stronger negative association with temperature. High mortality at lower temperature has some implications. In a tropical country with a short winter season and limited resources like Matlab, houses in rural areas are mostly roofed and walled with corrugated iron sheets. Particularly in the winter, the night temperatures inside and outside the house are the same. The cold wave accompanied by chill and fogs makes people ill as many do not have enough warm clothes. The short-term solution is to provide warm clothes to vulnerable groups, shifting them to nearby buildings for the time being and treating any illness. The long-term solution is socioeconomic development to enable houses to be built that can control temperatures.

Effect of rainfall on mortality might be more indirect. Cholera and non-cholera diarrhea peak pre-monsoon (April–May) and post-monsoon (September–October). Flooding, due to heavy rainfall, may be associated with an increase in diarrhea cases during the post-monsoon period (10), whereas high temperatures and long hours of sunshine (absence of rainfall) might explain the pre-monsoon peak (11). The pre and post-monsoon diarrhea peaks correspond with the higher mortality levels in infants and children aged 1–4 years old.

Small but significant heat effects on daily mortality in Matlab were found and it may be partly due to the absence of extreme heat waves or ‘heat island’ in rural environments. Large numbers of water bodies, trees, greenery fields and lower population density may make the relation weak (12). The possibility of gradual acclimatization/adaptation to hot weather in tropical conditions cannot be ruled out. Communities naturally adapt – physiologically, culturally and behaviorally – to living in warmer climates. Some evidence of adaptation was provided by previous studies. Minimum mortality temperature defined as the temperature of the lowest temperature-associated mortality observed in a city, was higher in the southern warmer cities than in the cooler northern cities in the United States (13). Alternatively, lower proportion of elderly population and cardiovascular deaths in this study population than in high-income urban cities may have resulted in weak heat effects on all-cause mortality; elderly and cardiovascular deaths have been generally sensitive to high temperature in previous studies in urban cities.
